# Whole Brain Monosynaptic Input of Distinct Neurons in the Globus Pallidus Externa

**DOI:** 10.1111/cns.70459

**Published:** 2025-06-04

**Authors:** Ming‐feng Ma, Jie Hu, Ya‐xin Hao, Kai‐ying Zhang, Xiang Zhang, Meng‐chu Zhu, Zong‐lei Zhou, Xiang‐shan Yuan, Fang Yuan

**Affiliations:** ^1^ Department of Neurobiology Hebei Medical University Shijiazhuang Hebei China; ^2^ Department of Anatomy and Histoembryology School of Basic Medical Sciences, Fudan University Shanghai China; ^3^ School of Nursing Hebei Medical University Shijiazhuang Hebei China; ^4^ Center for Medical Research and Innovation Shanghai Pudong Hospital, Fudan University Pudong Medical Center Shanghai China; ^5^ Department of Epidemiology School of Public Health, Fudan University Shanghai China; ^6^ Hebei Key Laboratory of Neurophysiology Shijiazhuang Hebei China; ^7^ The Key Laboratory of Neural and Vascular Biology, Ministry of Education Hebei Medical University Shijiazhuang Hebei China

**Keywords:** afferent inputs, FoxP2, globus pallidus externa, PV

## Abstract

**Objective:**

The globus pallidus externa (GPe) is involved in mediating physiological functions and contains two types of neurons: Forkhead box protein P2‐expressing (GPe^FoxP2^) neurons which inhibit motor, and parvalbumin‐expressing (GPe^PV^) neurons which improve motor. The functional complexity of the GPe, directly linked to its neuronal heterogeneity, necessitates exploring the neuroanatomical circuits of its distinct neuron types as a foundation for functional research.

**Methods:**

In this study, we employed specific, modified rabies viruses and adeno‐associated viruses to investigate the monosynaptic inputs of GPe^FoxP2^ and GPe^PV^ neurons.

**Results:**

We found that the input projections to both types of neurons are widespread, including the cortex, subcortical structures, amygdala, thalamus, hypothalamus, and brainstem. These inputs exhibit significant similarity, with 49 nuclei simultaneously innervating both types of neurons. However, GPe^PV^ neurons receive a lower proportion of inputs from the cortex and a higher proportion of inputs from the thalamus, compared to GPe^FoxP2^ neurons. Clustering analysis indicates that GPe^FoxP2^ neurons receive extensive afferent inputs from four nuclear clusters in the brain, whereas GPe^PV^ neurons receive afferent inputs from only three clusters, suggesting GPe^FoxP2^ neurons may be involved in more diverse functional regulations than GPe^PV^.

**Conclusion:**

Collectively, our results reveal the similarities and differences in the input projections to the two types of neurons in the GPe and lay the neuroanatomic groundwork for further studies to explore the critical physiological functions of GPe.

## Graphical abstract

Our results reveal the similarities and differences in the input projections to the two types of GABAergic neurons in the globus pallidus externa (GPe) and lay the neuroanatomic groundwork for further studies to explore the critical physiological functions of GPe.

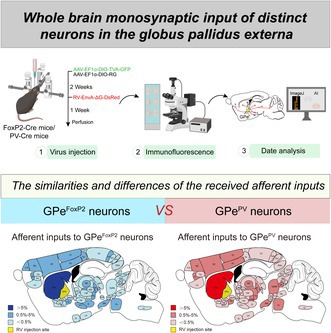

## 1 Introduction

The globus pallidus externa (GPe) is a significant component of basal ganglia circuitry and has a unique and powerful functions of motor information processing [1], as well as physiological functions such as regulating motor control [2], sleep [3], addictive behaviors [4], and states of consciousness [5]. In recent years, GPe has garnered considerable attention as a potential therapeutic target for various clinical conditions, including Parkinson's disease (PD) [6], Huntington's disease [7], epilepsy [8], etc. In PD, the over‐inhibition of the GPe by GABAergic neurons from the caudate putamen (CPu) is considered a key factor in the manifestation of motor symptoms [9]. However, whether the diverse functions of the GPe are regulated by other complex anatomical connections inside and outside the basal ganglia remains poorly understood.

GPe is a heterogeneous nucleus comprising various types of neurons, predominantly GABAergic [10]. The majority are parvalbumin (PV)‐expressing neurons, constituting 55%, while another subset expresses the transcription factor Forkhead box P2 (FoxP2), making up 30% [11, 12]. These two types of neurons not only exhibit significant differences in firing patterns but also show variations in their roles in physiological functions [3]. In a mouse model of PD, specifically enhancing the activity of GPe^PV^ neurons can improve motor function [6]. Interestingly, research has revealed that optogenetic excitation of GPe^FoxP2^ neurons exerts a strong inhibitory effect on movement [13], and the axon terminals of these neurons are primarily distributed in the CPu. Recent studies demonstrated that inhibition of GPe^PV^ neurons led to an increase in non‐rapid eye movement sleep [3], while GPe^FoxP2^ neurons exhibited a significant decrease in firing rate following the transition from wake to non‐rapid eye movement sleep [14]. The functional complexity of GPe is directly linked to its neuronal heterogeneity. To comprehensively decipher the function of GPe, it is necessary to first explore the neuroanatomical circuits of different types of neurons in GPe, laying the foundation for functional research.

Previous studies have shown that the GPe mainly receives a substantial amount of GABAergic afferent fibers from the CPu and glutamatergic afferent fibers from the subthalamic nucleus (STh) [15]. Additionally, previous studies have summarized that the GPe integrates various inputs from multiple systems, including the cortex, basal ganglia, thalamus, limbic system, and the brainstem [1]. However, these conclusions are drawn from diverse methodologies applied to various neuron types within the GPe. Classic retrograde tracers, such as cholera toxin B, Fluorogold, and horseradish peroxidase, effectively reveal the afferent inputs to the GPe; however, these tracers lack the specificity required to target individual neurons, offering only a generalized mapping of the GPe. Similarly, the use of conventional anterograde tracers to label axon terminals in the GPe from other nuclei is also hindered by issues of non‐specificity and inherent limitations.

For this reason, a retrograde monosynaptic tracking system based on rabies virus and Cre/loxP transgenic system [16] can effectively identify specific afferent connections of neurons. Here, we utilized two Cre‐dependent adeno‐associated viruses to express TVA and glycoprotein within the GPe of FoxP2‐Cre and PV‐Cre mice, followed by the use of a modified rabies virus to enable a more precise study of the monosynaptic afferent inputs to the GPe^FoxP2^ and GPe^PV^ neuronal populations. This foundational anatomical mapping paves the way for more in‐depth exploration of the neural circuits that underlie the distinct physiological functions of various neuron types.

## 2 Materials and Methods

### 2.1 Animals

Neural tracer experiments were performed in five FoxP2‐Cre mice (stock No. 030541, Jackson Laboratory), five PV‐Cre mice (stock No. 008069, Jackson Laboratory), and three wild‐type littermate mice (adult male mice, 10–12 weeks, 25–28 g). FoxP2‐Cre mice and PV‐Cre mice can express Cre recombinase under the control of their respective gene promoters. Animals were housed in individual cages at a constant temperature (22°C ± 0.5°C) and relative humidity (60% ± 2%) in a circadian rhythm with an automatically controlled 12:12 light: dark cycle (lights on at 7:00 a.m., lights off at 7:00 p.m.), with adequate food and water. All animal studies were conducted in accordance with the protocol approved by the Ethics Committee for Animal Experiments, School of Basic Medical College, Fudan University (License No. 20190221‐028). Every effort was made to minimize the number of animals used and any pain or discomfort experienced by the subjects.

### 2.2 Viruses and Surgeries

All viruses were packaged and purchased from BrainVTA (BrainVTA Co. Ltd., Wuhan, China) and stored at −80°C before use. The titer of rabies virus (RV‐EnvA‐ΔG‐dsRed) was 2 × 10^8^ genome copies/mL, and the Cre‐dependent AAV vector carrying rabies RG (AAV‐EF1α‐DIO‐RG) and TVA (AAV‐EF1α‐DIO‐TVA‐GFP) was packaged into 2/9 serotypes with a titer of 2 × 10^12^ genome copies/mL.

Mice were anesthetized with 1.5% isoflurane via continuous delivery with a mask and fixed onto a stereotaxic apparatus (RWD life science, Shenzhen, China) for all stereotaxic viral injections. The two helper viruses AAV‐EF1α‐DIO‐TVA‐GFP and AAV‐EF1α‐DIO‐RG were mixed at a ratio of 2:1 before virus injection, and 50 nL AAV mixture was injected unilaterally (10 nL/min) into GPe (coordinates: anterior–posterior = −0.3 mm, medial‐lateral = −2.5 mm, dorsal‐ventral = −3.0 mm) [3]. After injection, the pipette was placed again for 10 min to allow the virus to spread from the injection site, and then slowly withdrawn. Two weeks later, RV‐EnvA‐ΔG‐dsRed (100 nL) was delivered into the same position. After subcutaneous injections of Carprofen (5 mg/kg) with sterile saline, mice were warmed until recovery. We kept each mouse under close observation for another 7 days, such as weight, behavior, eating, and drinking activities. All mice were perfused for immunostaining.

### 2.3 Histology

One week after rabies virus injection, mice were deeply anesthetized with 50 mg/kg pentobarbital sodium (i.p.), followed by perfusion of saline in the heart and observation of the liver turning pale white, followed by perfusion of 100 mL of 4% paraformaldehyde (0.1 M PB, pH 7.4, ice cold). Next, the brain tissue was quickly removed from the skull and placed in 4% paraformaldehyde at 4°C for 12–16 h and then cryopreserved in 0.1 PB (pH 7.4) with 10%, 20%, and 30% sucrose at 4°C until they sank. Tissues were embedded with OCT compounds and stored in a −80°C freezer. Brains were sectioned coronally at 25 μm thickness on a cryostat (Leica CM1950) in three series and collected in 0.01 M phosphate‐buffered saline (PBS, pH 7.4). Every one‐third section was coverslipped with DAPI Fluoromount‐G mounting medium (Southern Biotech, 0100‐20).

To confirm the start cell in the GPe after virus injection, we immunostained the sections containing the GPe with PV and FoxP2 primary antibodies, respectively. Brain sections containing the GPe were incubated with the Goat anti‐PV primary antibody (1:2000, catalog number: PVG213, Swant, RRID: AB_2650496) and Goat anti‐FoxP2 primary antibody (1:500, catalog number: sc‐21069, Santa Cruz, RRID: AB_2107124) in 0.01 M PBS containing 5% bovine serum albumin and 0.25% Triton X‐100 at 4°C. After incubation at 4°C for 24 h, the tissue sections were combined with fluorescent secondary antibodies (Donkey anti‐Goat IgG Alexa 647, 1:1000, catalog number: 705‐605‐003, Jackson ImmunoResearch) and incubated in the dark at room temperature for 2 h. All antibodies were diluted with blocking solution. The brain slices were then washed three times with PBS and covered with DAPI Fluoromount‐G media.

### 2.4 Imaging and Data Analysis

The whole brain slices were imaged at 10× magnification using a virtual microscope slide scanning system (Olympus, Japan). A confocal microscope was used to obtain 40× enlarged images of brain sections to obtain more details (Leica, Germany). Based on the mouse brain atlas, combined with DAPI staining and the special anatomical structure of the brain, the brain slices were matched with the standard mouse brain atlas of the fourth edition of George Pessinos to ensure the accuracy of the location of dsRed‐labeled neurons, and the Adobe Illustration 2020 (Adobe System, San Jose, CA, USA) was applied to match the single nucleus. Open the image in ImageJ, convert from RGB mode to grayscale mode, and set a threshold to display dsRed‐labeled neurons, semiautomatically quantify the number of neurons and the density of cell bodies.

For statistical analysis, the data were imported into Prism8 (RRID: SCR_002798; GraphPad) was used for statistical analysis. All data are presented as mean ± SEM. No data outliers were excluded from this study. Data normality was evaluated using Shapiro–Wilk tests, with statistical approaches (parametric/nonparametric) selected according to distribution characteristics. Nonparametric analyses were applied to clustered z‐scores of monosynaptic input patterns. Multigroup comparisons of non‐normally distributed data used Kruskal–Wallis tests; where significant differences were found, pairwise comparisons were carried out with Dunn's post hoc tests, with *p* adjusted using the Bonferroni method. Divergent inputs to distinct neuronal populations were compared via multiple unpaired *t*‐tests (parametric analyses), with Benjamini‐Hochberg FDR correction. Statistical significance was considered at *p* < 0.05.

### 2.5 Hierarchical Clustering

Hierarchical clustering, a commonly used data clustering method by measuring between‐group similarity along with K‐means clustering analysis. It can employ the matrix of Euclidean distance or correlation coefficients as inputs. This process began with the random selection of K centroids, followed by calculating the nearest sample points to these centroids. The centroids were iteratively updated, thereby gradually partitioning the samples into K distinct categories. Specifically, the afferent input data received by GPe^FoxP2^ and GPe^PV^ were initially normalized using the log1p function in R software to calculate Z‐scores, which are commonly employed to reduce the influence of outliers. Afterwards, we assessed the similarity between the data by calculating the correlation of the number of projections from brain regions to the two types of neurons with the Pearson correlation test. Subsequently, to ensure a robust and reliable clustering result, we determined the optimal number of clusters (K value) based on the total within‐cluster sum of squares (WSS) and the Gap statistic. The WSS reflects the total dispersion of observations within each sample, while the Gap statistic evaluates whether the actual clustering effect is significantly different from random clustering. Collectively, a smaller within‐cluster sum of squares and a larger Gap value indicate a better clustering result. After clustering using a predefined K value, a heatmap with a color gradient was created to illustrate the strength of correlation, thereby revealing a general pattern regarding the number of incoming neurons provided by various brain nuclei. Additionally, the Z‐scores of each brain nuclei at an individual level were plotted in a heatmap based on the resulting clustering order, using distinct colors to visually represent the characteristics of inputs received by the two types of neurons.

## 3 Results

### 3.1 Strategies for Tracing Monosynaptic Inputs to Different GPe Neurons via Rabies‐Virus System

**FIGURE 1:**
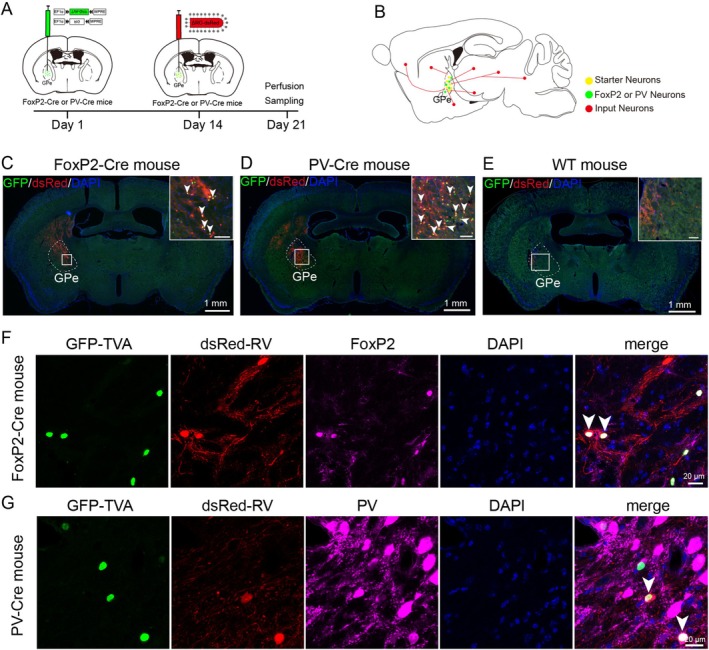
Experimental strategies for tracing monosynaptic inputs to GPe^FoxP2^ and GPe^PV^ neurons. (A) Schematic of experimental design. (B) Schematic of the starter cell in the GPe and their presynaptic inputs. (C–E) Representative images showed that after injection of helper virus and rabies virus in FoxP2‐Cre (C) and PV‐Cre (D) mice, there were neurons expressing GFP and dsRed in GPe, but not in wild‐type mice (E). Scale bar, 1 mm. (F, G) Fluorescence confocal images showed that the starter cells (yellow, co‐expressing EGFP and dsRed) infected with AAV helper virus and RV were also FoxP2‐positive in FoxP2‐Cre mice (F) and PV‐positive in PV‐Cre mice (G). Green, neurons affected by helper AAV; Red, neurons affected by rabies virus; Blue, DAPI‐stained nuclei; Magenta, FoxP2 or PV neurons. Scale bar, 20 μm.

To display input connectivity patterns of two types of GABAergic neurons of the GPe in the whole brain, we employed a cell type specific RV strategy in FoxP2‐Cre and PV‐Cre mice, respectively. First, two Cre‐dependent helper viruses (AAV‐EF1α‐DIO‐TVA‐EGFP and AAV‐EF1α‐DIO‐RG) were injected into the unilateral GPe of different Cre mice to synchronously express EGFP‐TVA and rabies glycoprotein (RG). Two weeks later, the attenuated and modified RV virus was injected into the same area of FoxP2‐Cre and PV‐Cre mice, respectively (Figure 1A). RV only infected neurons expressing TVA receptors on the FoxP2 neurons or PV neurons and spread retrogradely into upstream neurons that innervate FoxP2 neurons or PV neurons expressing RG after the second microinjection. One week after the second injection, the mice were perfused and brains were harvested for subsequent analysis (Figure 1A,B). After sectioning the tissues for observation and aligning them with the mouse brain atlas, we detected EGFP‐TVA and dsRed signals in the unilateral GPe of FoxP2‐Cre or PV‐Cre mice (Figure 1C,D). The initiating cells were defined as dual‐labeled neurons co‐expressing EGFP and dsRed signals in the GPe. Combined with FoxP2 or PV immunostaining, we demonstrated that almost all initiating cells were also colocalized with FoxP2 or PV signal, respectively (FoxP2 83.24% ± 3.81% vs. PV 82.76% ± 4.24%, Figure 1F,G). These results confirmed that initiating cells are FoxP2 neurons or PV neurons in the FoxP2‐Cre mice or PV‐Cre mice. For each group, the location of the vast majority of initiating cells was restricted to the GPe, although we found a very small number of EGFP and dsRed double‐positive neurons in the CPu. A number of control experiments were conducted by the same viral strategy in the GPe of wild‐type mice to define the specificity of the rabies virus tracing approach. This treatment demonstrated slight local background infection by RV was observed (Figure 1E), most likely due to the leaky expression of a low level of TVA. In contrast, all non‐specifically dsRed labeled neurons were only found near the injection sites within the GPe. Therefore, to exclude the impact of this nonspecifically labeling on the mapping of long‐range inputs, the following analysis excluded data originating from the GPe. Collectively, these findings confirm the efficacy of our tracing system in mapping long‐range monosynaptic inputs to distinct neuronal populations within the GPe. Moreover, the labeled neurons observed outside the injection sites validate their role as monosynaptic inputs to projection neurons in the GPe.

### 3.2 Presynaptic Input Patterns Onto GPe^FoxP2^ and GPe^PV^ Neurons

**FIGURE 2:**
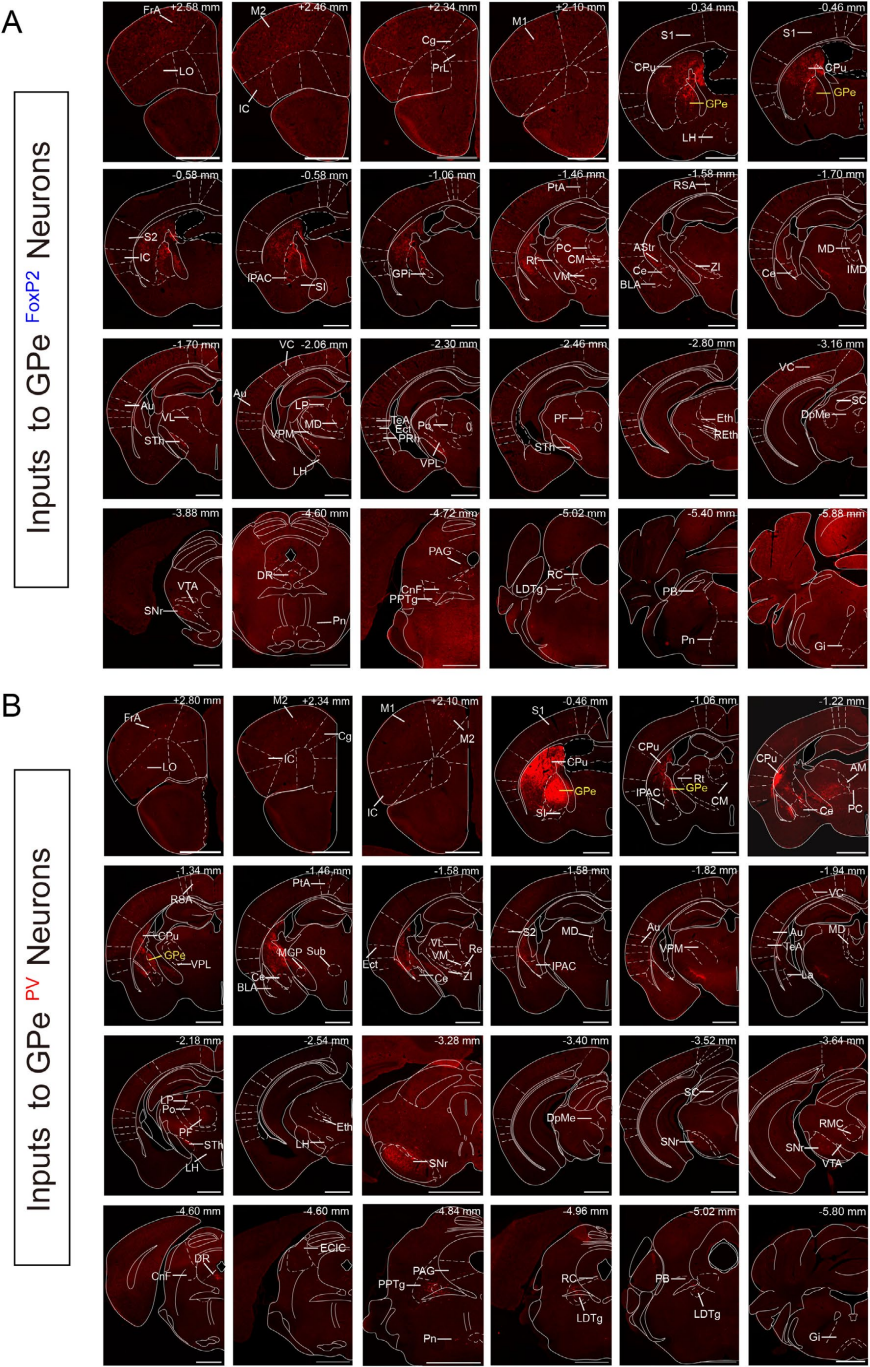
Representative images of monosynaptic inputs to GPe^FoxP2^ and GPe^PV^ neurons from the whole brain. Regions are labeled according to the Mouse Brain Atlas. Coronal slices of whole‐brain input from GPe^FoxP2^ (A) and GPe^PV^ (B) neurons. Only the ipsilateral aspect of the injection site is shown. Scale bar, 1 mm.

**FIGURE 3:**
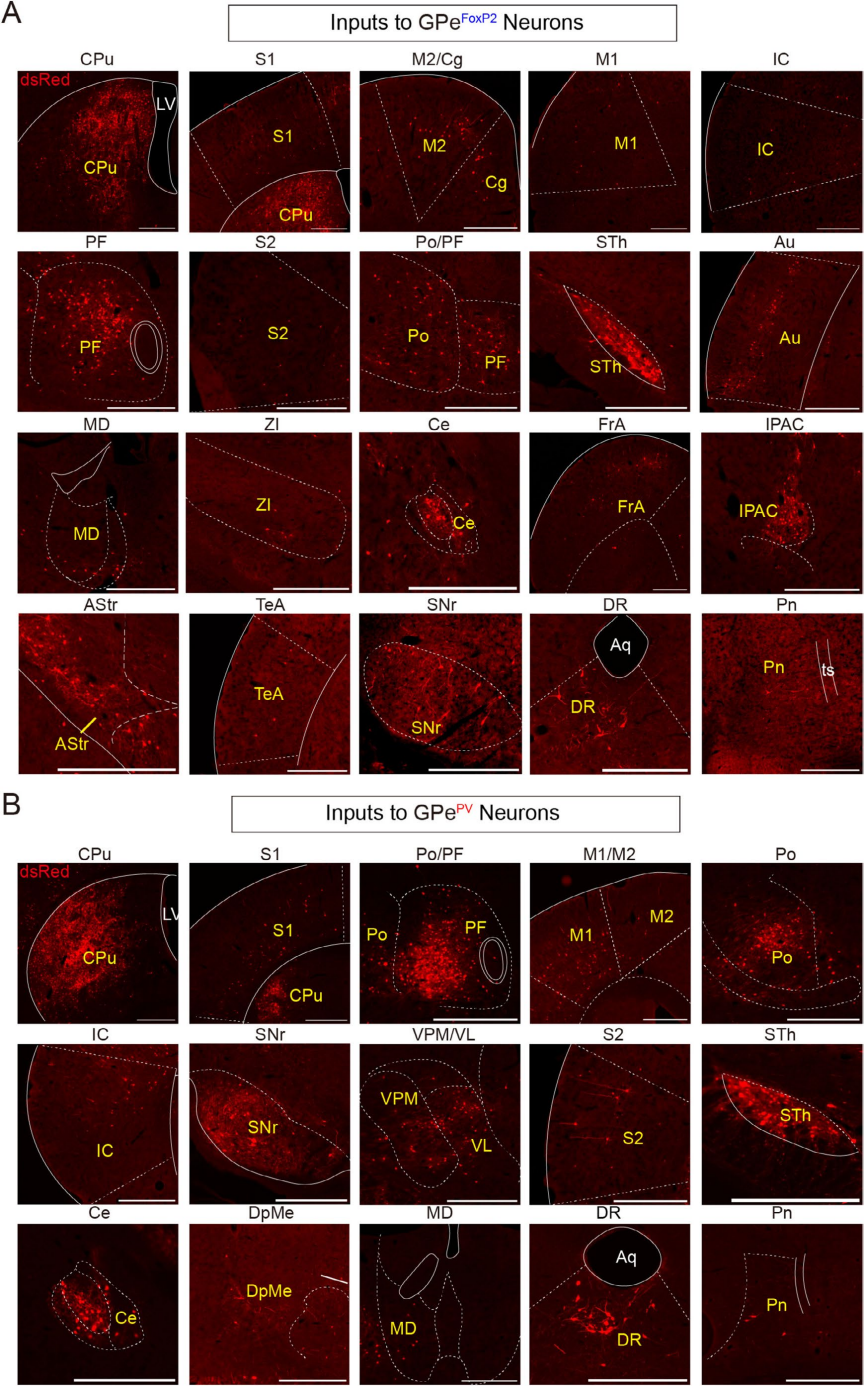
Representative images of selected brain regions with monosynaptic inputs to GPe^FoxP2^ (A) and GPe^PV^ (B) neurons. Scale bars, 0.5 mm.

**FIGURE 4:**
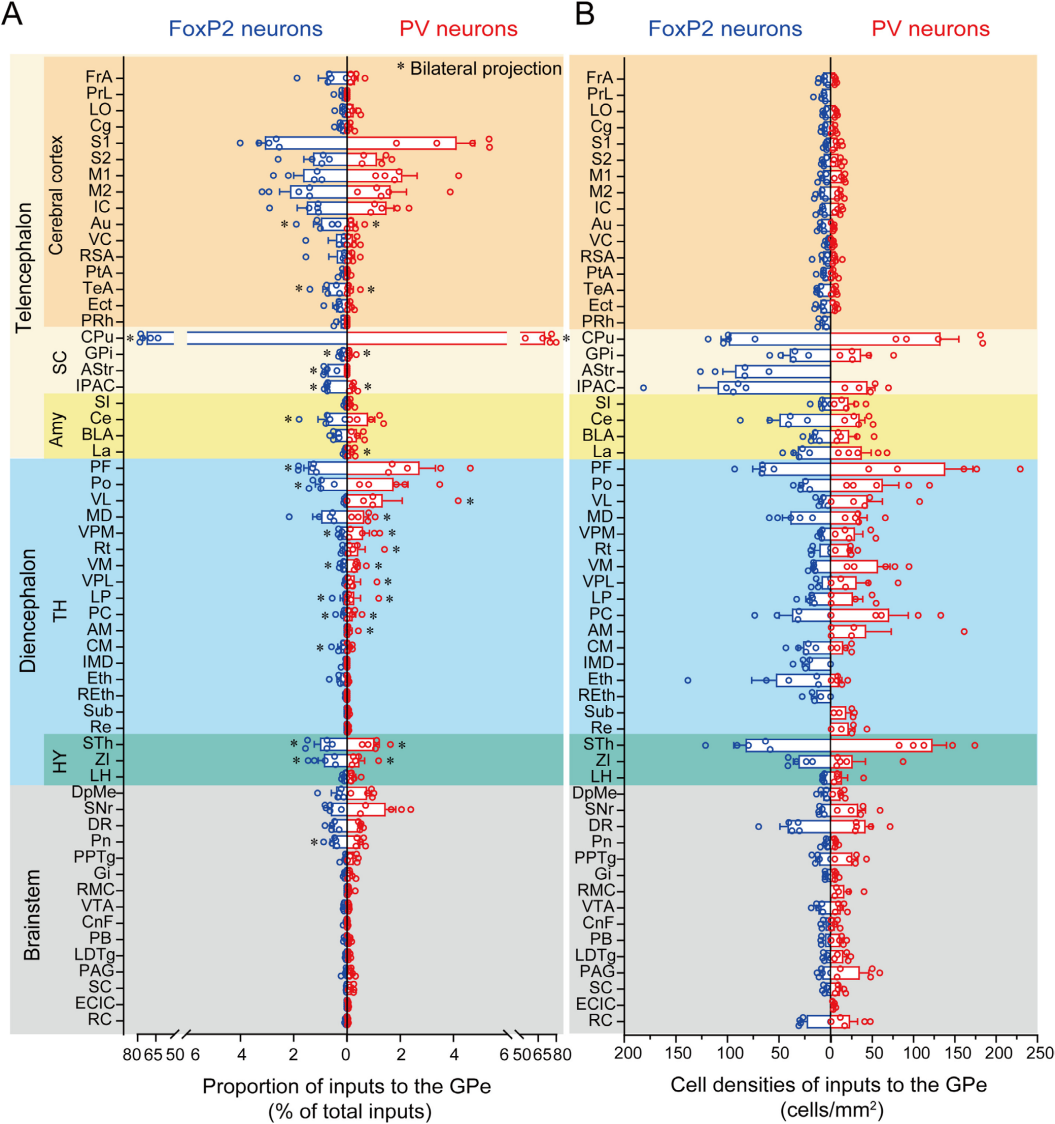
Summary of monosynaptic inputs to GPe^FoxP2^ and GPe^PV^ neurons. Quantitative analysis normalized proportions (A) and cell densities (B) of whole‐brain afferent nuclei of GPe^FoxP2^ and GPe^PV^ neurons. Error bar represents the SEM (*n* = 5). Brain areas are grouped into six general structures: the cerebral cortex, subcortical structures, and amygdala in the telencephalon; thalamus and hypothalamus in the diencephalon; and brainstem. Asterisks represent the bilateral inputs.

To investigate the distribution of monosynaptic inputs to GPe^FoxP2^ and GPe^PV^ neurons, we examined sequential coronal brain sections in five FoxP2‐Cre and PV‐Cre mice, respectively. Representative brain coronal sections from transsynaptic input tracing to FoxP2‐Cre mouse (Figures 2A and 3A) and PV‐Cre mouse (Figures 2B and 3B) demonstrate the distributions of dsRed‐labeled neurons in the whole brain. We identified brain regions containing dsRed‐labeled neurons as nuclei with monosynaptic connections to the two neuron types. The dsRed‐labeled input neurons of both GPe^FoxP2^ and GPe^PV^ neurons exhibited an exceptionally widespread and similar distribution, spanning along with rostral‐caudal axis, and were located in six major brain regions: the cortex, subcortical structures, and amygdala in the telencephalon; the thalamus and hypothalamus in the diencephalon; and the brainstem (Figure 4A and Table 1). Presynaptic dsRed‐labeled neurons to GPe^FoxP2^ neurons were observed in 54 nuclei throughout the brain, with bilateral labeling in multiple brain nuclei, including: the auditory cortex (Au) and temporal association cortex (TeA) in the cortex; CPu, globus pallidus internus (GPi), amygdalostriatal transition area (AStr), and interstitial nucleus of the posterior limb of the anterior commissure (IPAC) in subcortical structures; central amygdaloid nucleus in the amygdala; parafascicular thalamic nucleus (PF), posterior thalamic nuclear group (Po), ventral posteromedial thalamic nucleus (VPM), ventromedial thalamic nucleus (VM), lateral posterior thalamic nucleus, paracentral thalamic nucleus, and central medial thalamic nucleus (CM) in the thalamus; STh and zona incerta (ZI) in the hypothalamus; and pontine nuclei in the brainstem (Figure 4A).

Additionally, GPe^PV^ also receives afferent inputs from 54 brain regions, many of which are bilaterally labeled, such as Au and TeA in the cortex; CPu, GPi, AStr, and IPAC in subcortical structures; lateral amygdaloid nucleus in the amygdala; ventrolateral thalamic nucleus (VL), mediodorsal thalamic nucleus (MD), VPM, reticular thalamic nucleus, VM, ventral posterolateral thalamic nucleus, lateral posterior thalamic nucleus, paracentral thalamic nucleus, and anteromedial thalamic nucleus in the thalamus; and STh and ZI in the hypothalamus (Figure 4A). Interestingly, we found that GPe^FoxP2^ (Figure 2A) and GPe^PV^ (Figure 2B) receive afferent projections from 49 same nuclei within the total input nuclei, representative images of typical subregional inputs on the ipsilateral side of the injection sites were selectively displayed. To illustrate the predominant distribution of dsRed‐labeled presynaptic neurons across the brain, we displayed the representative and enlarged images of inputs (Figure 3). Here, we selected nucleus regions where the somatic proportion exceeded 0.5% as the primary sources of afferent input for presentation.

**TABLE 1:** Abbreviations and classifications of brain structures.

Abbreviation	Name	Parent brain region
AM	Anteromedial thalamic nucleus	Thalamus
AStr	Amygdalostriatal transition area	Subcortex
Au	Auditory cortex	Cortex
BLA	Basolateral amygdaloid nucleus, anterior part	Amygdala
Ce	Central amygdaloid nucleus	Amygdala
Cg	Cingulate cortex	Cortex
CM	Central medial thalamic nucleus	Thalamus
CnF	Cuneiform nucleus	Brainstem
CPu	Caudate putamen (striatum)	Subcortex
DpMe	Deep mesencephalic nucleus	Brainstem
DR	Dorsal raphe nucleus	Brainstem
ECIC	External cortex of the inferior colliculus	Brainstem
Ect	Ectorhinal cortex	Cortex
Eth	Ethmoid thalamic nucleus	Thalamus
FrA	Frontal association cortex	Cortex
Gi	Gigantocellular reticular nucleus	Brainstem
GPi	Globus pallidus internus	Subcortex
IC	Insular cortex	Cortex
IMD	Intermediodorsal thalamic nucleus	Thalamus
IPAC	Interstitial nucleus of the posterior limb of the anterior commissure	Subcortex
La	Lateral amygdaloid nucleus	Amygdala
LDTg	Laterodorsal tegmental nucleus	Brainstem
LH	Lateral hypothalamic area	Hypothalamus
LO	Lateral orbital cortex	Cortex
LP	Lateral posterior thalamic nucleus	Thalamus
M1	Primary motor cortex	Cortex
M2	Secondary motor cortex	Cortex
MD	Mediodorsal thalamic nucleus	Thalamus
PAG	Periaqueductal gray	Brainstem
PB	Parabrachial nucleus	Brainstem
PC	Paracentral thalamic nucleus	Thalamus
PF	Parafascicular thalamic nucleus	Thalamus
Pn	Pontine nuclei	Brainstem
Po	Posterior thalamic nuclear group	Thalamus
PPTg	Pedunculopontine tegmental nucleus	Brainstem
PRh	Perirhinal cortex	Cortex
PrL	Prelimbic cortex	Cortex
PtA	Parietal association cortex	Cortex
RC	Raphe cap	Brainstem
Re	Reuniens thalamic nucleus	Thalamus
REth	Retroethmoid nucleus	Thalamus
RMC	Red nucleus, magnocellular part	Brainstem
RSA	Retrosplenial agranular cortex	Cortex
Rt	Reticular thalamic nucleus	Thalamus
S1	Primary somatosensory cortex	Cortex
S2	Secondary somatosensory cortex	Cortex
SC	Superior colliculus	Brainstem
SI	Substantia innominata	Subcortex
SNr	Substantia nigra, reticular part	Brainstem
STh	Subthalamic nucleus	Hypothalamus
Sub	Submedius thalamic nucleus	Thalamus
TeA	Temporal association cortex	Cortex
VC	Visual cortex	Cortex
VL	Ventrolateral thalamic nucleus	Thalamus
VM	Ventromedial thalamic nucleus	Thalamus
VPL	Ventral posterolateral thalamic nucleus	Thalamus
VPM	Ventral posteromedial thalamic nucleus	Thalamus
VTA	Ventral tegmental area	Brainstem
ZI	Zona incerta	Hypothalamus

### 3.3 Analysis of Input Neurons Innervating GPe^FoxP2^ and GPe^PV^ Neurons

To reveal the differences of afferent projections, the proportion and cell density of dsRed‐labeled neurons in individual brain regions were analyzed to quantitatively describe the whole‐brain distribution of afferent inputs to GPe^FoxP2^ and GPe^PV^ neurons. Statistical analysis reveals that the CPu of basal ganglia provides the majority of inputs to GPe^FoxP2^ (72.75%) and GPe^PV^ (70.76%) neurons (Figure 4A), confirmed the previous results that adenosine 2A receptor neurons of the CPu formed directly synapse with neurons of the GPe including PV and FoxP2 neurons [3]. At the same time, using specific tracing methods, we further confirmed the differential distribution of afferent inputs from the CPu to two types of neurons within the GPe. GPe^FoxP2^ neurons receive more inputs from the dorsomedial striatum, while GPe^PV^ neurons receive more inputs from the dorsolateral striatum (Figure 3). We defined nuclei with over 1% of upstream labeled neurons as major afferent regions. We found that GPe^FoxP2^ neurons receive primary inputs from the CPu (72.75%) in the basal ganglia, primary somatosensory cortex (S1) (3.09%), secondary motor cortex (M2) (2.15%), primary motor cortex (M1) (1.65%), insular cortex (IC) (1.51%), secondary somatosensory cortex (S2) (1.28%) in various cortical areas, PF (1.47%) and Po (1.02%) in the thalamus, and STh (1.01%) in the hypothalamus. In contrast, GPe^PV^ neurons receive the majority of their inputs from the CPu (70.76%) in the basal ganglia, S1 (4.13%), M1 (2.09%), M2 (1.65%), IC (1.49%), and S2 (1.13%) in various cortical areas, PF (2.73%), Po (1.76%), and VL (1.35%) in the thalamus, STh (1.03%) in the hypothalamus, and the reticular part of substantia nigra (SNr) (1.46%) in the brainstem (Figure 4A).

In the statistical analysis of somatic count percentages and densities within upstream nuclei, we organized the data along the anterior–posterior axis of the coronal plane. The analysis reveals that both neuronal types predominantly receive afferent inputs from regions in the telencephalon and diencephalon. While both neuron types exhibit similarities in their afferent inputs, GPe^FoxP2^ neurons receive a higher proportion of cortical inputs (14.44%) compared to GPe^PV^ neurons (12.15%). This trend persists across subcortical structures, with GPe^FoxP2^ receiving 74.53% of inputs versus 71.25% for GPe^PV^, as well as in the hypothalamus (2.02% vs. 1.76%, respectively) (Figure 4A). Conversely, GPe^PV^ neurons receive a greater proportion of inputs from the thalamus (9.07%) compared to GPe^FoxP2^ neurons (4.99%), a pattern mirrored in the brainstem (4.43% for FoxP2 vs. 2.67% for PV) (Figure 4A). These results are further corroborated by the somatic density analysis of upstream nuclei (Figure 4B).

**FIGURE 5:**
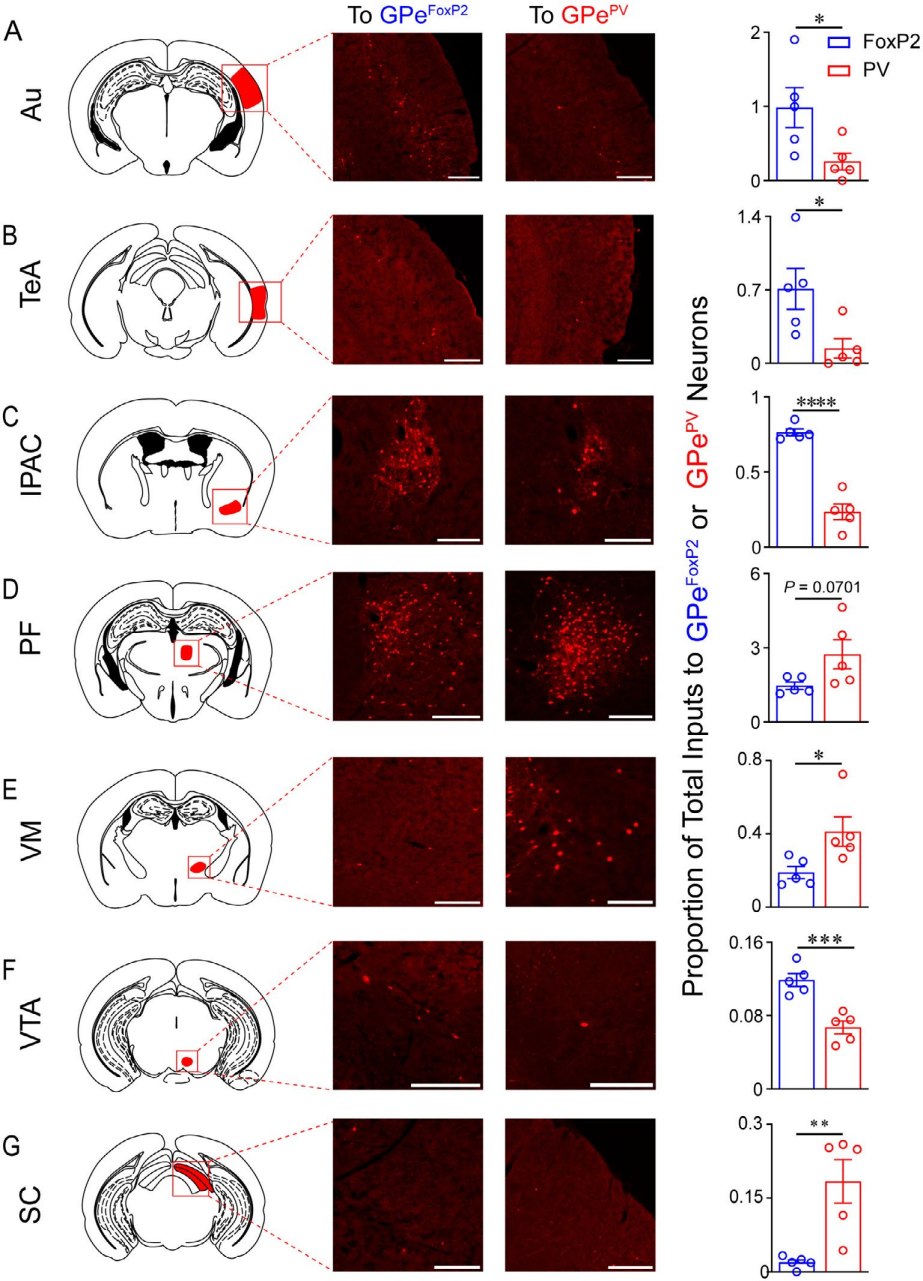
Representative images of nuclei with differential projections to GPe^FoxP2^ and GPe^PV^ neurons. The right‐most columns indicate quantification of dsRed+ cells in each nucleus (*n* = 5 each, unpaired *t*‐test). Error bars represent SEM. *p < 0.05, **p < 0.01, ***p < 0.001, ****p < 0.0001. Scale bar, 200 μm.

Furthermore, we conducted a differential analysis of the percentage data for each afferent input nucleus of the two neuron types and compared the magnified images of each nucleus. Through analysis, it can be determined that the afferent inputs of the two types of neurons differ in the following nuclei: Au and TeA in the cortex, IPAC in the subcortical structures, PF and VM in the thalamus, and ventral tegmental area and superior colliculus in the brainstem (Figure 5). Remarkably, the differential afferent inputs from these specific nuclei closely align with the overall comparison between the two neuron types across all regions of the cortex, subcortical structures, thalamus, and brainstem. This finding reinforces the idea that both neuron types receive afferent inputs from across the brain, exhibiting both similarities and distinctions. These results suggest that the physiological roles of these neurons may differ depending on the functional brain regions involved.

**FIGURE 6:**
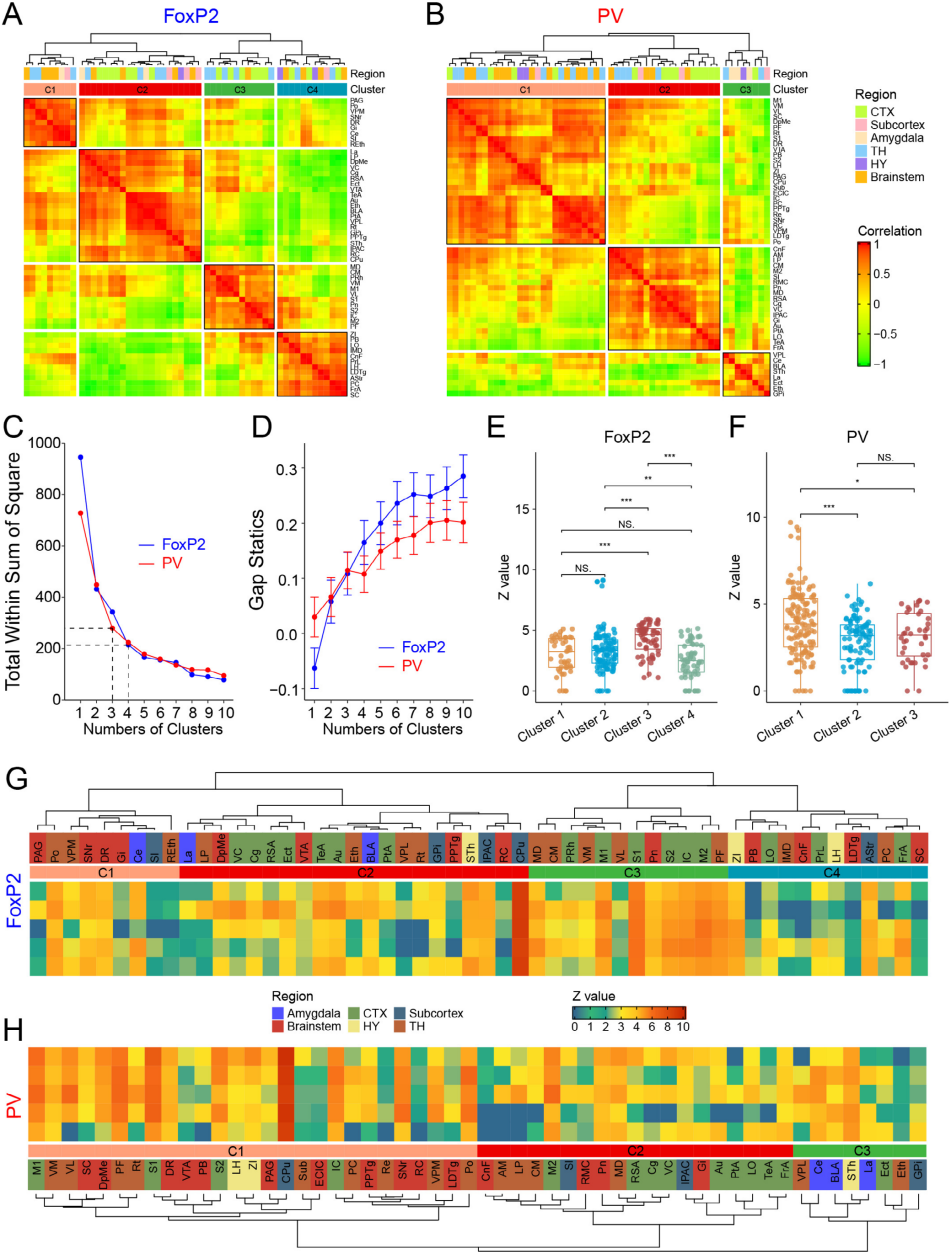
Comparison of Whole‐Brain Monosynaptic Input Patterns of GPe^FoxP2^ and GPe^PV^ Neurons. (A, B) Hierarchical clustering based on correlation distance matrices of projection neuron count‐related data across brain regions. The intensity of color in the heatmap indicates the correlation of projection neuron counts between pairs of nuclei within each brain region, with red indicating a positive correlation and green indicating a negative correlation. (C, D) The number of clusters was determined by the Gap statistic and the total within‐cluster sum of squares, with *K* = 4 and *K* = 3 used for FoxP2 and PV, respectively. (E, F) Box plots represent the normalized cluster distributions, illustrating the Z‐scores of clusters under the monosynaptic input patterns of GPe^FoxP2^ and GPe^PV^ neurons (Kruskal–Wallis/Dunn's test (Bonferroni)). Error bars represent SEM. *p < 0.05, **p < 0.01, ***p < 0.001. (G, H) Based on the clustering order of brain regions shown in panels A and B, the heatmaps feature the normalized neuron counts projecting to GPe^FoxP2^ and GPe^PV^ neurons for each brain region across animals.

To investigate the distribution patterns of incoming inputs received by GPe^FoxP2^ and GPe^PV^ neurons from various brain nuclei, and to uncover potential functional correlations, we conducted a correlation analysis of the cell count and hierarchical clustering of the 54 upstream nuclei projecting to GPe^FoxP2^ and GPe^PV^. Before the clustering analysis, we conducted pairwise correlation analyses based on the log‐normalized number of somas received by GPe^FoxP2^ and GPe^PV^ from 54 brain nuclei across the whole brain, in order to verify the validity of the data and assess their similarity (Figure 6A,B). Subsequently, we explored the change in the value of WSS and Gap statistic over the number of clusters. For nuclei projecting to GPe^PV^, we found a notable decrease in the WSS values as the number of clusters increased from 1 to 3, and the curve generally tended to flatten if the number of clusters was greater than 4. Also, the Gap statistic exhibited a similar trend as WSS, and the indicator slightly decreased when brain nuclei were grouped into 4 clusters. Similar observations were also detected for nuclei projecting to GPe^FoxP2^, where WSS values decreased significantly and the Gap statistic drastically increased until the number of clusters reached 4. Notably, although detailed subgrouping helps to delineate the nucleus‐specific characteristics, while it does not necessarily enable the discovery in the homogeneity of various nuclei since we also attempted to unravel brain nuclei that perform similar biological functions. Therefore, taking overall consideration, we adopted *K* = 4 and *K* = 3 for the clustering analysis under the administration of GPe^FoxP2^ and GPe^PV^, respectively (Figure 6C,D). In the preserved order of brain regions following hierarchical clustering, the Z‐scores of each nucleus for each mouse were presented in the heatmaps (Figure 6G,H). Furthermore, we examined the distribution characteristics of the total normalized Z‐scores for each cluster. GPe^FoxP2^ neurons received extensive inputs from the brain, with four clusters (Figure 6A,E). The overall Z‐scores in cluster 3 had the highest overall Z‐score among the four clusters (Figure 6E). The nuclei in cluster 3 include several nuclei of the thalamus (MD, CM, VM, VL, and PF, Figure 6A) and cortex (perirhinal cortex, M1, M2, S1, S2, and IC, Figure 6A), suggesting that cluster 3 of GPe^FoxP2^ neurons is primarily responsible for receiving and integrating more concentrated information transmitted from both the thalamus and cortex. In contrast, GPe^PV^ neurons received relatively less inputs from the brain, with three clusters (Figure 6B,F). The nuclei in cluster 1 contained 27 nuclei, accounting for half of the total number from different brain regions, including cortex, thalamus, subcortex, and brainstem (Figure 6B), suggesting that cluster 1 of GPe^PV^ neurons is mainly responsible for receiving and integrating more extensive information from the entire brain. In addition, given the differences in the number of clusters between GPe^FoxP2^ and GPe^PV^ neurons, it can be inferred that GPe^FoxP2^ neurons have greater functional diversity, as they form four clusters with relatively similar sizes. In contrast, GPe^PV^ neurons exhibit lower heterogeneity, as they are divided into only three clusters, with the first cluster accounting for half of the nuclei. By analyzing the two clusters with the highest Z‐scores in both GPe^FoxP2^ and GPe^PV^, we can observe the validity of the clustering and the potential functional differences between these two types of neurons in different physiological functions.

**FIGURE 7:**
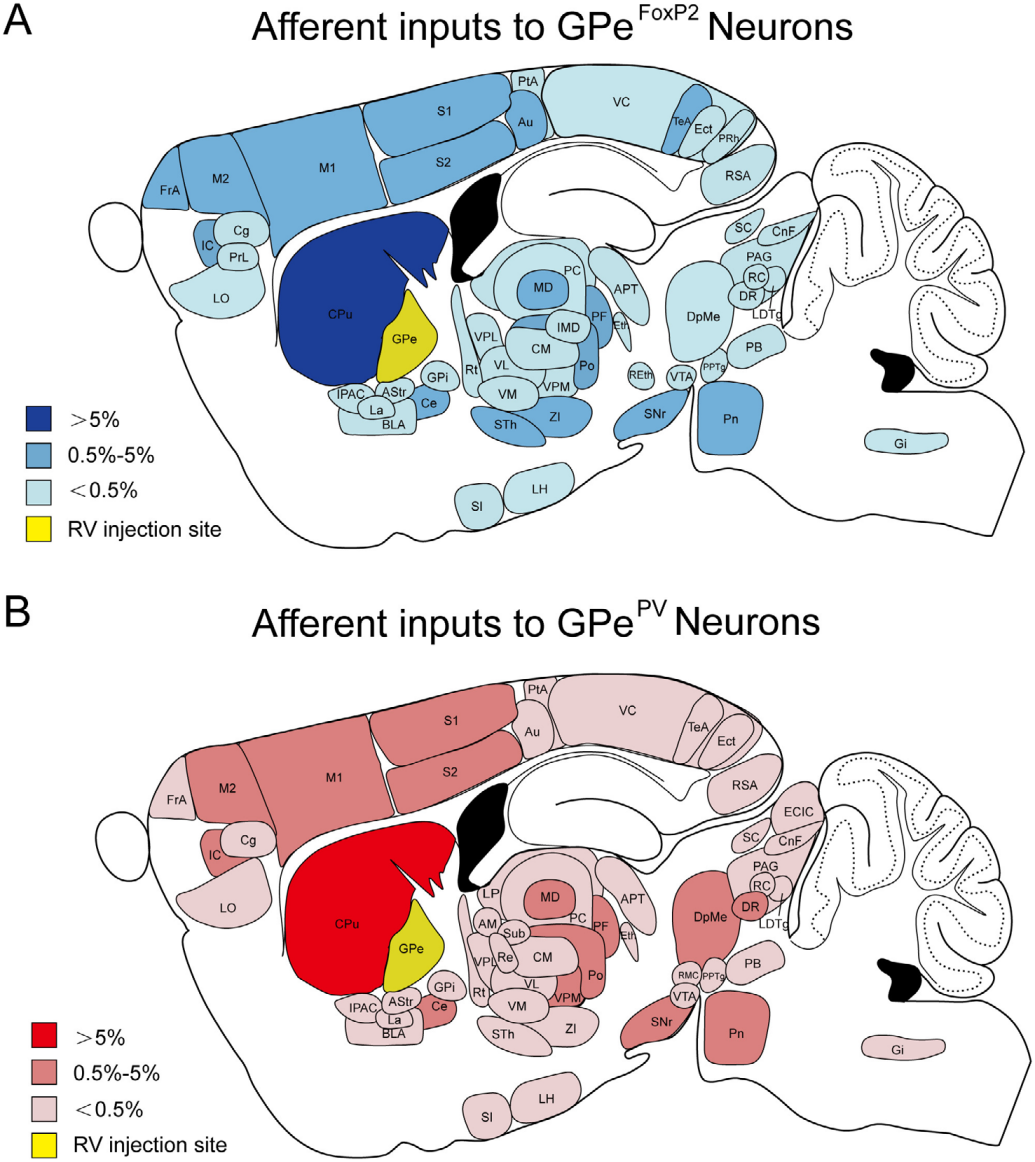
Summary of the major afferent inputs onto GPe^FoxP2^ and GPe^PV^ neurons. Brain regions that afferently project onto GPe^FoxP2^ (A) and GPe^PV^ (B) neurons are shown on a schematic sagittal section. The color transparency represents the percentage of afferent inputs onto GPe^FoxP2^ (blue) and GPe^PV^ (red) neurons.

To visually compare the input neurons of GPe^FoxP2^ and GPe^PV^ neurons, we mapped the monosynaptic inputs of the entire brain and displayed the proportion of input neurons in each nucleus (Figure 7). The color shades indicate that the majority of afferent neurons in the brain are mostly located in the CPu. In addition, the differences between the two in the thalamus, cortex, and other regions can be observed more intuitively, which is more conducive to analyze the physiological functions of this nucleus.

## 4 Discussion

As a key structure within the basal ganglia, the GPe plays a pivotal role in the regulation of physiological functions. To gain deeper insight into the neural circuitry mechanisms by which GPe^FoxP2^ and GPe^PV^ neurons contribute to physiological processes, we utilized a highly specific RV‐mediated retrograde strategy, enabling comprehensive and precise mapping of their distinct inputs. Our findings reveal the similarities and diversities in the presynaptic inputs to GPe^FoxP2^ and GPe^PV^ neurons from diverse brain regions and provide an anatomical basis for exploring the distinct neural circuits underlying their specific physiological functions.

### 4.1 Comparison With Previous Retrograde Tracing Studies

The GPe serves as a relay station in the basal ganglia and is considered a therapeutic target for diseases such as PD [17]. Its neural connectivity has been a subject of ongoing interest. The classical model posits that the GPe functions as an intrinsic nucleus within the basal ganglia circuit, receiving inputs mainly from the CPu and STh [1]; however, growing evidence indicates that the GPe also receives afferent inputs from multiple regions outside the basal ganglia. In previous studies, traditional tracing methods, such as classical retrograde tracers and anterograde tracers, have revealed that the GPe receives afferent inputs from structures including the cortex, basal ganglia, thalamus, limbic system, and the brainstem [1, 18, 19, 20, 21]. Furthermore, it is well known that the GPe is a heterogeneous nucleus, comprising distinct neuronal populations with different neuronal markers, including PV and FoxP2 [11]. Consequently, traditional tracing methods are insufficient to identify the specific inputs to GPe^FoxP2^ and GPe^PV^ neurons.

Our viral strategy involves the intracranial injection of modified RV and AAV into the GPe of FoxP2‐Cre and PV‐Cre mice, aiming to precisely label distinct neurons without the limitations of traditional tracers. This approach utilizes transgenic mice to represent FoxP2 and PV neurons, offering a comprehensive understanding of the anatomical connections throughout the brain through quantitative analysis. Based on our current transsynaptic retrograde tracing results, we found that both GPe^FoxP2^ and GPe^PV^ neurons receive the most afferent inputs from the CPu, consistent with previous study [22]. Previous studies have shown that the STh is one of the primary sources of glutamatergic input to the GPe [15]. Optogenetic and whole‐cell voltage clamp recordings reveal that, in terms of input strength, the projection from STh to PV neurons is significantly stronger than that to Npas1‐expressing (Npas1+) neurons [23]. Notably, FoxP2 is expressed in 60% of Npas1+ neurons of the GPe [12]. However, our current findings reveal that the percentage difference in afferent inputs from the STh to GPe^FoxP2^ and GPe^PV^ neurons is not particularly substantial. This phenomenon may be attributed to their diverse requirements in function, structure, connectivity, information processing capabilities, and evolutionary and developmental contexts. This could also explain why the number of GPe^PV^ neurons is nearly twice that of GPe^FoxP2^ neurons, despite both types of neurons receiving inputs from the same number of nuclei. Although previous studies have used retrograde tracers such as fluorescent beads to detect the afferent inputs received by the GPe, which includes inputs from corticotropin releasing factor neurons in the bed nucleus of the stria terminalis and the hypothalamus paraventricular nucleus, our findings did not reveal such afferent inputs to GPe^FoxP2^ and GPe^PV^ neurons [24]. These findings suggest that the bed nucleus of the stria terminalis and hypothalamus paraventricular nucleus may primarily regulate other neurons within the GPe, such as corticotropin‐releasing factor receptor 1 neurons, which may be involved in the stress response of the central autonomic nervous system [24, 25].

Our clustering analysis indicates that the incoming inputs to GPe^PV^ and GPe^FoxP2^ can be categorized into three and four clusters, revealing potential functional differences. This suggests that the role of GPe^PV^ neurons may be somewhat specialized, primarily involved in motor regulation, while GPe^FoxP2^ neurons may be involved in a broader range of physiological functions, such as the effects induced by ethanol consumption and anxiety‐related behaviors [24, 26]. Similarly, our analysis shows that GPe^FoxP2^ neurons receive more cortical input (such as TeA and Au), while GPe^PV^ neurons are predominantly influenced by thalamic input (such as PF and VM). The TeA encodes contextual memory, decision‐making, and cross‐modal integration, while the Au is involved not only in auditory processing but also in motor control [27, 28, 29]. The abundant cortical input enables GPe^FoxP2^ neurons to integrate a variety of sensory information, thereby supporting more complex activities in the brain. The interaction between the basal ganglia and the thalamus plays a pivotal role in motor control, with both PF and VM involved in regulating movement [30]. The thalamic input to GPe^PV^ neurons likely allows them to focus on motor control [31]. The differential input patterns from the cerebral cortex and thalamus to the distinct types of neurons in the GPe reflect the functional divergence and complementarity of these neurons. At the same time, our clustering analysis results further support the functional differences between GPe^FoxP2^ neurons and GPe^PV^ neurons.

We have validated previous studies indicating that the GPe receives afferent inputs from various regions, including the cortex (M1, M2, S1, S2, and prefrontal cortex), the basal ganglia (CPu, STh, SN, and GPi), the thalamus (PF, VL, VM, VPM, Po, and ZI), the limbic system (central amygdaloid nucleus, lateral hypothalamic area, and substantia innominata), and the brainstem (superior colliculus, periaqueductal gray, dorsal raphe nucleus, and mesencephalic locomotor region (MLR)) [1]. Previous studies have predominantly explored this through neuron tracing techniques in mice. However, fMRI studies conducted on right‐handed female subjects revealed that the GPe receives inputs from both the CPu and STh [32]. Furthermore, DBS‐fMRI on normal rats showed that the GPe not only receives inputs from these two nuclei but also from the ZI [33]. These findings have been further validated and refined by our results, providing additional support for the anatomical connections of the GPe. Additionally, we identified several previously unreported afferent inputs to these two types of neurons in the GPe. These inputs originate from diverse brain regions, including cortical areas such as the lateral orbital cortex, cingulate cortex, Au, visual cortex, retrosplenial agranular cortex, parietal association cortex, TeA, ectorhinal cortex, and perirhinal cortex. Subcortical sources include the amygdala (e.g., lateral amygdaloid nucleus), the thalamus (e.g., intermediodorsal thalamic nucleus, ethmoid thalamic nucleus, retroethmoid nucleus, submedius thalamic nucleus, and reuniens thalamic nucleus), and subcortical structures like AStr and IPAC. Additional inputs were identified from brainstem regions, including the magnocellular part of the red nucleus, parabrachial nucleus, external cortex of the inferior colliculus, and raphe cap. These findings offer valuable insights into the potential functional contributions of the GPe within these broader neural circuits, as the physiological functions associated with these nuclei are diverse, including roles in fear processing (e.g., AStr) [34], reward (e.g., IPAC) [35], and short‐term memory (e.g., Au) [36]. The afferent inputs from these newly identified nuclei are relatively sparse, a phenomenon we suspect is due to the enhanced sensitivity of the retrograde tracing system employing the modified rabies virus. Compared to previous conventional tracing techniques, our retrograde trans‐synaptic tracing results exhibit greater specificity and sensibility relative to particular microcircuit findings. However, given that the genetically modified mice used in this study were all male, we recognize the potential influence of sex on anatomical circuits and plan to explore this factor in greater detail in future studies.

Cholinergic neurons in the GPe account for approximately 2%–5%, while other studies have confirmed that these cholinergic neurons belong to the nucleus basalis of Meynert, which is located between the ventral aspect of the GPe and internal capsule [37, 38]. A study has cataloged the afferent inputs to cholinergic neurons in the nucleus basalis of Meynert, revealing that the inputs to these neurons share 30 common nuclei with two types of GABAergic neurons in the GPe [39]. Both groups receive the largest proportion of inputs from the CPu. However, a notable distinction is that these cholinergic neurons receive significantly more incoming inputs from the amygdaloid region [39]. The primary input station and output nucleus in the basal ganglia are the CPu and SNr, respectively. Previous studies utilizing the same retrograde tracing technique have investigated the incoming inputs to these two nuclei, offering valuable insights into the overall afferent inputs to the basal ganglia. Compared to the GPe, neurons in the CPu, including dopamine receptor type 1 (D1) and dopamine receptor type 2 (D2) receive a larger volume of afferent inputs from the cortex (approximately 50%). More surprisingly, GABAergic neurons in the GPe, along with the two types of neurons in the CPu, share incoming inputs from over 20 common brain regions [40]. Additionally, the SNr receives a greater volume of afferent inputs from the basal ganglia, with the largest contribution coming from the CPu [41]. This reveals that the nuclei within the basal ganglia are intricately interconnected anatomically, yet exhibit distinct differences, offering new clues for understanding their roles in motor, cognitive, and other functions.

Studying the afferent inputs received by GPe^FoxP2^ and GPe^PV^ neurons across the whole brain not only anatomically elucidates the potential mechanisms underlying some of these known functions, but also provides an anatomical foundation and direction for further research into physiological responses and diseases in which the GPe may be involved.

### 4.2 Effects of GPe Distinct Neurons on Motor Control

The cortex–basal ganglia–thalamus–cortex circuit serves as a cornerstone of the motor control loop, playing a pivotal role in regulating movement. The cortex initiates motor commands, which are transmitted from the basal ganglia to the thalamus through direct and indirect pathways. The activity of the direct pathway ultimately facilitates motor initiation by the cerebral cortex, primarily through the CPu‐GPi/SNr circuit within the basal ganglia [42]. In contrast, the indirect pathway, consisting of the CPu‐GPe‐STh‐GPi/SNr route, exerts an inhibitory effect on cortical activity [42]. The GPe, situated at the core of the indirect pathway within the basal ganglia, plays a crucial role in regulating and coordinating movement. Studies have shown that abnormal activity of GPe neurons is highly correlated with motor dysfunctions in PD patients [43], and high‐frequency deep brain stimulation of the GPe in human patients, as well as prolonged optogenetic activation of GPe^PV^ neurons in dopamine‐depleted mice, has been demonstrated to alleviate motor symptoms of PD [44, 45]. Interestingly, increasing the activity of GPe^FoxP2^ neurons has been shown to reduce the activity of striatal neurons and inhibit CPu‐dependent motor behaviors [14]. These findings suggest that both types of GABAergic neurons within the GPe are related to motor regulation. Moreover, as an output nucleus of the basal ganglia, the SNr plays a crucial role in the regulation of sleep and motor control [41]. Previous studies have confirmed that both types of neurons in the GPe can project to the SNr [12], and our results demonstrate that the SNr can simultaneously innervate to PV and FoxP2 neurons within the GPe. This reciprocal microcircuit between the GPe and SNr suggests a precise regulatory mechanism within the basal ganglia for motor control.

Beyond its traditional role in transmitting motor information within the basal ganglia, accumulating evidence suggests that the GPe directly receives motor input from the cortex, brainstem, and thalamus. The M1 serves as a critical hub for processing external stimuli and facilitating the integration of sensory inputs into motor outputs [46]. Both GPe^FoxP2^ and GPe^PV^ neurons receive substantial afferent input from M1 and the M2. Moreover, the brainstem, acting as a communication nexus, plays a pivotal role in maintaining posture to support motor activities [47]. Within this context, the MLR emerges as a key area coordinating motor output with spinal executive motor networks [48]. Notably, the cuneiform nucleus and pedunculopontine tegmental nucleus, two principal components of the MLR, provide direct input to GPe^FoxP2^ and GPe^PV^ neurons. Furthermore, the thalamus, a central relay for motor control, integrates cortical signals and transmits motor commands to the basal ganglia and brainstem to initiate movement [49]. Several thalamic nuclei contribute to motor processes, including, but not limited to, the VL, MD, and PF [30, 50, 51]. Among these, the PF provides the most extensive afferent input to GPe^FoxP2^ and GPe^PV^ neurons. In addition to its motor functions, the GPe also integrates emotional and behavioral signals. The central amygdala, a critical region for emotion regulation, sends projections to the GPe. This amygdala‐GPe circuit is involved in processing unconditioned stimuli and modulating fear‐learning behaviors [24]. Inputs from various amygdala nuclei influence motor outputs, driving contextually appropriate escape and freezing behaviors during stress responses [24]. Emerging evidence underscores the GPe's role as a key pathway mediating the involvement of the medial subthalamic nucleus in escape and defensive behaviors [52]. These findings highlight the diverse functional repertoire of the GPe, extending beyond motor control to encompass complex integrative roles in behavioral and emotional regulation.

### 4.3 The Influence of Distinct Neurons in GPe on Goal‐Directed Behavior and Decision‐Making

Goal‐directed behavior refers to actions performed to achieve specific objectives, enabling adaptive decision‐making when there is a significant discrepancy between expected and actual outcomes. Emerging evidence indicates that the GPe plays a critical role in both goal‐directed and habitual behaviors. Optogenetic inhibition of dorsomedial striatum and dorsolateral striatum pathways projecting to the GPe has been shown to, respectively, promote goal‐directed and habitual behaviors [47]. Moreover, excessive activation of GPe neurons within the indirect pathway has been demonstrated to impair decision‐making in PD model mice [53]. These findings highlight the importance of investigating the afferent inputs to the GPe as a means to address clinical challenges in the treatment of PD.

Our research into the afferent inputs to GPe^FoxP2^ and GPe^PV^ neurons reveals that the GPe receives projections from multiple nuclei associated with goal‐directed and decision‐making behaviors. Specifically, GPe^FoxP2^ and GPe^PV^ neurons receive inputs from decision‐related regions, including the CPu, M2, IC, S2, and frontal association cortex [54, 55, 56, 57]. Notably, M2 is primarily involved in decision formation and motor planning, with its projections to the CPu modulating visual perception and decision‐making [54]. These nuclei, which are strongly interconnected with the GPe, provide compelling evidence for the GPe's involvement in decision‐making processes. Key regions implicated in goal‐directed behavior include the M1, CPu, and PF [58, 59, 60]. Among these, the CPu, a critical structure within the basal ganglia, is essential for making appropriate choices and facilitating goal‐directed behaviors [60]. Additionally, the PF, which regulates goal‐directed behavior, serves as the primary input to the CPu [59, 60]. Both the CPu and PF, in turn, provide substantial afferent inputs to the GPe. Together, these findings suggest that GPe^FoxP2^ and GPe^PV^ neurons may represent the principal neuronal populations through which the GPe exerts its influence on goal‐directed behavior and adaptive decision‐making. While this hypothesis requires further experimental validation, our findings provide a robust anatomical framework for investigating the neural circuitry underlying adaptive decision‐making and goal‐directed behaviors.

### 4.4 Effects of GPe Distinct Neurons on Processing Sensory Information

The cerebral cortex can be considered the most intricate and essential part of the human brain, serving as the final convergence area for various sensory information pathways, including visual, auditory, and somatosensory inputs such as touch and pain, while also participating in a wide range of physiological functions. Cortical information is generally transmitted through two pathways: the direct pathway (cortex‐CPu‐GPi/SNr) and the indirect pathway (cortex‐CPu‐GPe‐STh‐GPi/SNr), with additional studies suggesting the presence of a hyperdirect pathway [42]. An increasing body of evidence indicates a direct connection between the cortex and the GPe, providing a more efficient channel for regulating various physiological functions. For example, studies in rats have shown that the M1 and M2 regions can directly target the GPe, supplying additional excitatory input to the basal ganglia, thereby influencing various physiological functions through complex mechanisms [61]. Our specific retrograde tracing of two types of GABAergic neurons within the GPe revealed that GPe^FoxP2^ and GPe^PV^ receive direct afferent inputs from 16 and 14 cortical regions, respectively, offering compelling evidence for cortical input to the GPe. These regions include the motor cortex, somatosensory cortex, visual cortex, Au, and other brain areas that provide sensory information.

The cerebral cortex transforms processed sensory information into behavioral commands, with the somatosensory and motor systems working in close coordination during tactile exploration. Sensory signals can be transmitted via relay neurons in the brainstem and thalamus, reaching the motor cortex to induce movement [62]. As part of the basal ganglia, the GPe is involved in the integration and processing of sensory information [63]. It receives input from the cerebral cortex and influences thalamic activity through both the direct and indirect pathways, thereby modulating the transmission of sensory signals [63]. In terms of input, both types of neurons receive projections from multiple cortical areas, including S1, S2, suggesting that the GPe can directly receive sensory information from higher brain centers, thus facilitating better regulation of movement. Additionally, the GPe receives afferent inputs from various thalamic sensory‐related nuclei, such as the VPM and CM [64, 65]. The VPM processes somatosensory information [64], while the CM serves as an integration center for bodily and visceral sensations [65]. We also speculate that distinct GABAergic neurons in the GPe may act as a hub for processing sensory information and regulating movement, offering a novel perspective for future research in related fields.

These findings collectively indicate that the GPe is capable of receiving sensory input and transmitting motor information, thereby enhancing the coordination of motor control. They further enrich our understanding of the cortical‐thalamic‐basal ganglia circuits involved in motor control. They also provide valuable insights for exploring the potential functions of this nucleus in greater depth.

## 5 Conclusion

Overall, the whole‐brain monosynaptic retrograde tracing provides a precise and comprehensive description of the specific afferent inputs to GPe^FoxP2^ and GPe^PV^ neurons. We found that the majority of input nuclei to PV neurons and FoxP2 neurons in the GPe are the same; while the proportions of projections from some nuclei to these two types of neurons differ, suggesting both similarities and differences in their functions. Our findings establish a structural framework for the circuit mechanisms underlying specific physiological functions, further highlighting the pivotal role of PV and FoxP2 neurons in GPe‐regulated behaviors through neuroanatomical evidence.

## Author Contributions

Study design and manuscript drafting/revising: Fang Yuan, Xiang‐shan Yuan, and Zong‐lei Zhou. Running of the experiments, acquisition of the data, and figure drawing: Ming‐feng Ma, Jie Hu, Ya‐xin Hao, and Kai‐ying Zhang. Acquisition of the data and statistical analysis: Xiang Zhang and Meng‐chu Zhu. All authors have read and approved the final version of the manuscript.

## Conflicts of Interest

The authors declare no conflicts of interest.

## Data Availability

The data that support the findings of this study are available from the corresponding author upon reasonable request.
